# A statistical model for monitoring shell disease in inshore lobster fisheries: A case study in Long Island Sound

**DOI:** 10.1371/journal.pone.0172123

**Published:** 2017-02-14

**Authors:** Kisei R. Tanaka, Samuel L. Belknap, Jared J. Homola, Yong Chen

**Affiliations:** 1 School of Marine Sciences, University of Maine, Orono, Maine, United States of America; 2 Climate Change Institute, University of Maine, Orono, Maine, United States of America; 3 Department of Anthropology, University of Maine, Orono, Maine, United States of America; 4 School of Biology and Ecology, University of Maine, Orono, Maine, United States of America; Universidade de Aveiro, PORTUGAL

## Abstract

The expansion of shell disease is an emerging threat to the inshore lobster fisheries in the northeastern United States. The development of models to improve the efficiency and precision of existing monitoring programs is advocated as an important step in mitigating its harmful effects. The objective of this study is to construct a statistical model that could enhance the existing monitoring effort through (1) identification of potential disease-associated abiotic and biotic factors, and (2) estimation of spatial variation in disease prevalence in the lobster fishery. A delta-generalized additive modeling (GAM) approach was applied using bottom trawl survey data collected from 2001–2013 in Long Island Sound, a tidal estuary between New York and Connecticut states. Spatial distribution of shell disease prevalence was found to be strongly influenced by the interactive effects of latitude and longitude, possibly indicative of a geographic origin of shell disease. Bottom temperature, bottom salinity, and depth were also important factors affecting the spatial variability in shell disease prevalence. The delta-GAM projected high disease prevalence in non-surveyed locations. Additionally, a potential spatial discrepancy was found between modeled disease hotspots and survey-based gravity centers of disease prevalence. This study provides a modeling framework to enhance research, monitoring and management of emerging and continuing marine disease threats.

## Introduction

The American lobster (*Homarus americanus*), which is of critical economic and ecological importance throughout northeastern USA and Atlantic Canada, is currently being threatened by the emergence of shell disease. The shell disease in *H*. *americanus* is manifested as necrosis and lesions on the dorsal carapace of infected individuals that can result in decreased survival [1] and decreased reproductive success [2]. Shell disease in *H*. *americanus* was first reported in the 1930s, and various forms of lobster shell disease have been observed (e.g., endemic shell disease, impoundment shell disease, and diet-induced shell disease) [3–5]. Notably, shell degradation associated with disease decreases the market value of infected individuals, resulting in economic and market loss in this lucrative fishery [6,7].

Epizootic shell disease (ESD) is a recently observed degradation of the lobster cuticle by a suite of bacteria (e.g., *Aquimarina homaria*) [1]. Individual susceptibility to ESD has received increased research attention following the host susceptibility hypothesis proposed by Tlusty et al. [8]. This hypothesis states that the internal condition of a lobster ultimately determines whether an infection becomes established, with physiological stress likely being the strongest indicator of susceptibility. This notion was generally supported by subsequent studies evaluating the influence of water temperature [9], pollutants [1,10], and diet [5]. Additional studies of shell disease etiology noted significant shifts in microbial communities between the shells of infected and uninfected lobsters, suggesting importance of a polymicrobial, rather than single species, pathogen [11]. A major outbreak of ESD was first observed in Long Island Sound (LIS) in 1996, which was followed by the unprecedented rise and spread of ESD among Southern New England (SNE) lobster stocks. Prior to 1999, the lobster fishery in LIS was the third largest in the country, with landings valued at more than $35 million [12]. However, in 2013 the Atlantic States Marine Fisheries Commission (ASMFC) required the states surrounding LIS to take steps to reduce the total lobster harvest by 10 percent, resulting in the first-ever seasonal closure of the LIS lobster fishery [13]. Concern over the stability of the lobster fishery has forced many fishermen to abandon their traditional livelihoods and pursue new careers outside of the lobster industry [14,15].

Tools are required that will allow the fishery to deal with possible future spread of lobster shell disease. The development of a modeling framework that can provide (1) ecological interpretation of factors associated with disease prevalence, and (2) more reliable, contemporary disease maps at policy-relevant spatial scales has been advocated as an important step in understanding the harmful effects of oceanic diseases [16,17]. There are presently two broad types of modeling approaches available for predicting spatiotemporal disease prevalence: empirical-based statistical models that seek to quantify associations between disease prevalence and environmental factors (e.g., [18]) and process-based mechanistic models that seek to simulate biological or ecological processes that drive disease prevalence (e.g., [19]). It is generally acknowledged that both approaches can be used to facilitate proactive disease management.

The objectives of this study were to develop empirical-based statistical models to (1) quantify associations of lobster shell disease occupancy and abundance with environmental, spatial, and ecological factors, and (2) predict relative lobster shell disease prevalence in non-surveyed locations to provide a spatially-varying disease probability map across the entire study area to identify potential disease hotspots that remain undetected by the existing survey programs. We hypothesized that the spatial distribution of shell disease prevalence is associated with external factors such as salinity, water temperature, depth, distance offshore, sediment type, latitude and longitude, as well as host sex and life stages. To this end, a delta-generalized additive modeling (GAM) framework was developed to evaluate the relative contributions of a variety of environmental and biological factors to shell disease occupancy and abundance. GAMs have the advantage of reconciling highly non-linear and non-monotonic relationships that are common in nature, and can serve as either descriptive or predictive statistical models [20].

This study highlights the utility of pairing existing fishery-independent datasets with a non-parametric and parsimonious modeling approach to enhance the knowledge of how lobster shell disease associates with various abiotic and biotic factors. Ultimately, our findings will provide policy-relevant information for effective ecosystem-based marine disease surveillance programs, which could be of value for the U.S. lobster fishery.

## Materials and methods

### Case study area

**Fig 1:**
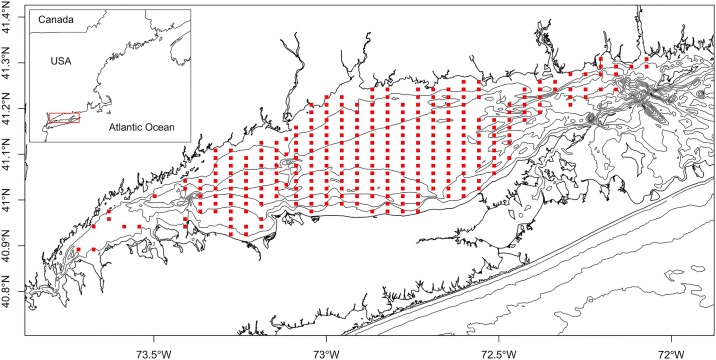
Sampling locations of the Long Island Sound bottom trawl survey used in this study (2001 to 2013). Each sampling site is 1.85*3.7 km.

The LIS is an estuary that is 182 km long and 33.8 km wide with an average depth of 22.6 m (Fig 1). The bathymetry of LIS is composed by four major basins with a maximum depth of 60.4 m. The LIS is weakly stratified as the salinity ranges from 23 ppt at the western end to 35 ppt at the eastern end [21]. Three major rivers (Thames, Housatonic, and Connecticut) account for the majority of freshwater input into LIS. Runoff and drainage along the coast of New York and Connecticut also deliver freshwater into the sound [22].

### Modeled data

**Fig 2:**
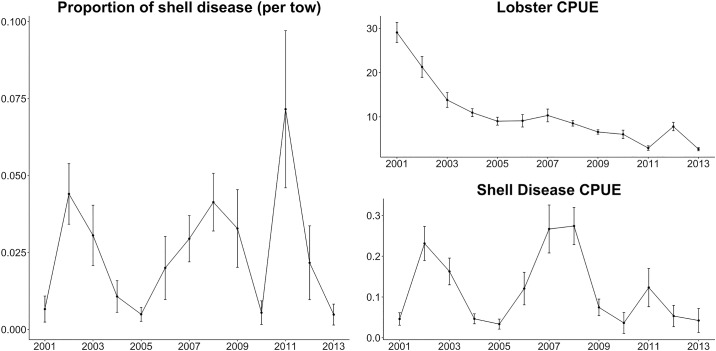
Abundance indices of American lobster (*Homarus americanus*) and shell disease per tow in Long Island Sound, USA. For calculation of American lobster CPUE see Tanaka and Chen (2015). CPUE: catch-per-unit-effort.

The lobster shell disease data were collected by bi-annual bottom trawl survey conducted by the Connecticut Department of Energy and Environmental Protection (CTDEP) during 2001 and 2013 (Fig 2). The CTDEP survey employs a stratified random design based on 12 strata (4 depth strata * 3 substrate strata). Samples were collected using a 14 m otter trawl with a 51 mm codend. Date, location, bottom temperature, bottom salinity, depth, and biological information of each lobster (carapace length (CL), sex, and shell disease presence) were recorded at each tow (Table 1). The survey area is divided into 1.85*3.7 km sites assigned to the 12 strata [23]. Spring surveys were conducted during the months of April- June, and fall surveys were conducted from September through October. *In situ* data are collected once a month from 40 sites that are randomly selected from within each stratum, resulting in a total of 200 sites annually. The survey was conducted at 3.5 knots for a targeted duration of 30 minutes during daylight hours to reduce sampling bias related to diurnal variability in catchability [24,25]. There were no changes associated with the size specification for the trawl equipment during the survey.

**Table 1:** A list of variables identified as candidate explanatory variables for delta generalized additive modeling approach with corresponding VIF value. All variables listed in this table were measured directly from the Long Island Sound bottom trawl survey (2001–2013).

Variables	Type	Description	VIF^b^
Season	Temporal	Season trawl was conducted: Spring = March-May, Fall = September-November	n/a
Year	Temporal	Year trawl was conducted	n/a
Latitude (Degree)	Spatial	Measurement of latitude trawl was conducted (mid trawl point)	2.2312
Longitude (Degree)	Spatial	Measurement of longitude trawl was conducted (mid trawl point)	1.9888
Distance Offshore (km)	Spatial	Measurement of distance between trawl location and coastline	1.5965
Depth (m)	Abiotic	Observed depth at trawl location	2.2378
Bottom Temperature (°C)	Abiotic	Observed bottom temperature at trawl location	1.4678
Bottom Salinity (ppt)	Abiotic	Observed bottom salinity at trawl location	1.7772
Stage	Biotic	Adult (CL^a^ > 60 mm) and Juvenile (CL < = 60 mm)	n/a
Sex	Biotic	Female and Male (unspecified sex were omitted)	n/a

^a^CL: Carapace Length.

VIF: Variance Inflation Factor.

The monitoring of lobster shell disease began in 2001, and a total of 1,246 tows that collected 18,322 lobsters were initially explored in this study. A tow was considered satisfactory for the analysis only when it recorded (1) number of shell disease-positive lobsters with relevant biological information (e.g. sex and carapace length), (2) geographical information (e.g. latitude and longitude), and (3) environmental information (e.g. bottom water temperature and salinity, depth). A total of 1,234 tows that collected 17,838 lobsters met these criteria were used for the analysis. The dataset showed an overdispersion of shell disease abundance due to the high number of tows that caught zero infected lobsters. A lobster was considered to be free of shell disease if the shell surface shows no signs of the disease (i.e. the default condition) or if the lobster had limited necrotic spots (e.g. pitting and “cigarette-like burn” mark on the shell surface) or lesions (e.g. damage that penetrates carapace to inner musculature). A visual inspection was conducted to identify shell disease on the claws, carapace, tail, and legs. A lobster was considered to be infected if more than 10% of shell surface shows signs of shell disease (e.g., pitting and lesions). Several types of lobster shell disease have been documented, which are not differentiated here. Despite our inability to distinguish among shell diseases, the condition we describe here is most likely ESD given its known prevalence throughout the study area [1,26–28].

The shell disease catch-per-unit-effort (CPUE) was considered to be a good indicator of lobster shell disease prevalence in the study area [29–31]. Survey-CPUE is a commonly used indicator for monitoring changes in relative abundance of fish stocks [32]. Studies have shown that CPUE is most reliable when the sampling units are homogeneous in their characteristics and operating procedure [32–34], and gravity centers of CPUE can be used to better understand the spatiotemporal dynamics of fish stocks [34–37]. A nominal shell disease CPUE at station *i*, in season *j*, and year *y* was calculated as;

$$CPUE_{i,j,y}=\left(\frac{Count_{i,j,y}}{TowDuration_{i,j,y}}\right)*20$$ (1)

where Count represents the total quantity of shell disease positive lobster caught. Tow duration varied between 20 to 30 minutes but was standardized to 20 minutes at each sampling station [31]. To analyze the spatial distribution of lobster shell disease, the longitudinal and latitudinal gravitational centers of nominal disease CPUE in year *y* were calculated by;

$$Lon_{y}=\frac{\sum_{i=1}^{K}\left(Lon_{i}*CPUE_{y,i}\right)}{\sum_{i=1}^{K}CPUE_{y,i}}$$ (2)

$$Lat_{y}=\frac{\sum_{i=1}^{K}\left(Lat_{i}*CPUE_{y,i}\right)}{\sum_{i=1}^{K}CPUE_{y,i}}$$ (3)

where *Lon*_*i*_ represents the longitudinal point of the station *i* between -73.63 and -72.07 E; *Lat*_*i*_ represents the latitudinal point of the station *i* between 40.92 and 41.31 N; *CPUE*_*y*,*i*_ denotes the nominal shell disease CPUE at station *i* in year *y*; *K* is the total number of stations.

### Generalized additive model

#### Model development

A delta (also known as Hurdle or Two-stage) generalized additive modeling (GAM) approach was applied to account for zero-inflation and overdispersion [38–40]. GAM is a semi parametric extension of the generalized linear model and commonly used in ecological studies [41,42]. GAMs assume that the response variables are independent, and use spline smooth function to define nonlinear relationships between the response and explanatory variables [20]. With the delta approach, occupancy and abundance observations are modeled separately to formulate the overall prediction of relative species abundance while it allows independent evaluation of predictor variables for both occurrence and abundance, which often differ [43,44].

Lobsters within each tow were grouped by stage (adult: >60 mm carapace length, juvenile: ≤60 mm carapace length) and sex (male and female), allowing every tow to have up to 4 groups of lobsters (2 stage * 2 sexes) [31,45,46]. This categorization technique developed by [39] can relate biological characteristics of a tow-subgroup to environmental information recorded by the corresponding tow. For each tow-subgroup, the delta-GAM separately modeled: (1) the “encounter rate probability” of shell disease (i.e. a proportion expressed as total number of shell disease positive lobsters divided by total number of lobsters), and (2) the “positive catch probability” of shell disease (i.e. number of shell disease positive lobsters conditional on presence). The general delta-GAM formulation can be written;

Encounter rate probability (*y*^1^):

$$logit\left(y\right)=\alpha +\overset{p}{\underset{i=1}{\sum }}f\left(x_{i}\right)+\varepsilon$$ (4)

Positive catch probability (*y*^2^):

$$\text{ln}\left(y\right)=\alpha +\overset{p}{\underset{i=1}{\sum }}f\left(x_{i}\right)+\varepsilon$$ (5)

Overall prevalence probability:

$$D=y^{1}*y^{2}$$ (6)

where *a* denotes an intercept term, *f* denotes the non-parametric cubic spline smooth function; x_i_ denotes the *i*^*th*^ explanatory variable directly measured by the CTDEP survey; and *ε* is the residual error term. The first stage GAM modeled the proportion of shell disease per tow-subgroup (i.e. encounter rate probability) using a logit link function and a binomial error distribution. Here, the total number of lobsters in each response variable served as a prior weight on the contribution of the data to the first stage GAM fitting procedure to account for the difference in response variable size. The second stage GAM modeled the shell disease abundance per tow-subgroup conditional on presence (i.e. positive catch probability) using a log link function and a negative binomial error distribution. The overall prevalence probability (*D*) was derived by multiplying the products from both stages [40,44].

Variance inflation factor (VIF) analysis with an acceptable value below 3.0 was conducted to minimize collinearity among candidate explanatory variables [41]. To avoid unnecessary model complexity and computation time, boosted regression tree (BRT) analysis was conducted for each GAM to incorporate candidate bivariate terms [44,47]. To prevent model overfitting, the maximum degrees of freedom was set at 5 (k = 5) for univariate terms and 30 (k = 30) for bivariate terms [42,44,48]. Furthermore, gamma = 1.4 was set for each GAM to place a heavier penalty on each term to prevent overfitting [42,49].

All statistical analyses were conducted in the R programming environment [50]. GAMs were built and fitted using the *mccv* package [51] and *fmsb* [52] and *dismo* [53] were used to implement VIF and BRT analyses.

#### Model selection and validation

Chi-square statistical significance tests and Akaike information criteria (AIC) were used as the model selection criteria. A stepwise backward selection was applied to identify an optimal model in each stage [52]. First, a full model was built for each stage using all of the candidate univariate and bivariate terms identified through VIF and BRT analyses. Second, the least statistically significant variable was removed using the specified p-value significance threshold (p < 0.05) [39,52–54]. Variable removal was conducted one at a time and the reduced model was refit to the data. Candidate univariate and bivariate terms were kept in the model if they contributed to a lower AIC [55]. The stepwise model selection procedure was repeated until an optimal model was identified according to the above criteria at each stage (i.e. a model with lowest AIC and included only significant variables). Finally, model diagnostic plots were examined to evaluate residual patterns and model assumptions.

A cross-validation study was conducted to evaluate the performance of the best-fitting delta-GAM [41]. A randomly selected subset representing 80% of the original data (training data) was used to develop and calibrate the delta-GAM, and the remaining 20% (testing data) was used to evaluate the model performance. The model predictions were compared to the observations and linear regression analysis was used to evaluate the model performance. The cross-validation process was repeated 100 times using a random partition in each step. The model performance was quantified by 100 sets of linear regression parameters: an intercept (*α*) closest to 0, a slope (*β*) closest to 1, and higher *R*^*2*^.

### Environmental data

Because a GAM does not generate coefficients that can be multiplied by conventional grid maps of the covariates, spatial predictions were made by constructing new environmental datasets of the study area [56]. Bottom temperature and salinity estimates by depth, time, and location in the study area were modeled by the Finite-Volume Community Ocean Model (FVCOM) runs from 2001 to 2013. FVCOM is an ocean circulation model developed by University of Massachusetts Dartmouth and Woods Hole Oceanographic Institution [57]. The FVCOM has been configured for the Northwest Atlantic Shelf region, with horizontal resolution ranging from 20 m in river mouths to as coarse as 10 km towards the open boundary off the shelf [57]. Bathymetry layers were obtained from the U.S. Coastal Relief Model [58]. The surficial substrate layer in LIS was obtained from the U.S. Geological Survey (resolution: 0.00001 decimal degrees or 1.11 m) [59]. Substrate classifications in included; gravel (pebbles defined as 2.00–64.00 mm, cobbles defined as 64–256 mm, boulder defined as above 256 mm), gravel-sand (0.62–2.00 mm), sand-clay (0.001–0.004 mm), silt (0.004–0.062 mm)/sand, sand- clay/silt, sand-silt/clay, and sand/silt/clay [60].

### Predictions of spatiotemporal patterns in shell disease prevalence

The shell disease prevalence predictions derived by the best-fitting delta-GAM were assigned to every FVCOM grid in the study area and universal kriging interpolation technique was used to produce high-resolution maps for interpretation [41,61,62]. This procedure was repeated for every year within the predictive capacity of the best-fitting delta-GAM (2001–2013). The spatial distribution of median GAM outputs was mapped to interpret the overall spatial variability in shell disease prevalence. The longitudinal and latitudinal gravitational centers of observed shell disease prevalence between 2001 and 2013 were compared to the modeled disease hotspots to evaluate magnitude of spatial discrepancy due to potential biases associated with the survey design and subsequent sample size.

## Results

### Significance of abiotic and biotic variables

A total of 2,008 tow-subgroups out of 1,234 tows were analyzed during the time period of 2001–2013 (n = 17,838 lobsters). Shell disease positive lobsters (n = 363) sampled in LIS ranged in size from 37.3 to 88.1 mm CL, with mean CL of 69.81 mm and median CL of 71 mm. The shell disease samples were collected at various depth ranges from 4.9–42.7 m and between 40.98:41.31°N and 73.37:72.07°W. The observed bottom temperature and salinity associated with shell disease positive lobster ranged from 3.9–22.1°C and 24.8–31.5 ppt respectively.

**Fig 3:**
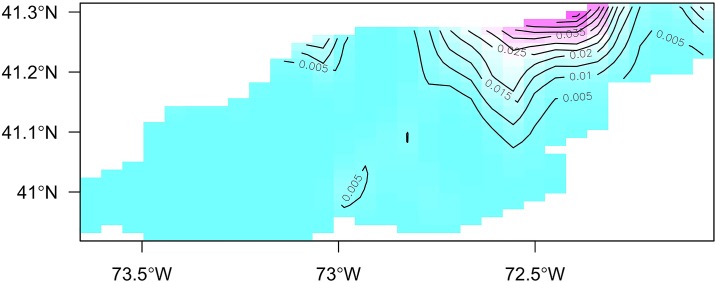
Partial generalized additive model (GAM) plot describing the significant interactive contribution of bivariate location variable in the best-fitting encounter rate probability model (1st stage).

The location variable identified as a bivariate interaction covariate by latitude and longitude was found to be the most important determinant in the probability of shell disease presence. The response surface of the location variable indicates that probability of shell disease presence increased toward the northeastern region of LIS (Fig 3). Neither longitude nor latitude was found to be significant in the best-fitting positive catch probability model.

**Fig 4:**
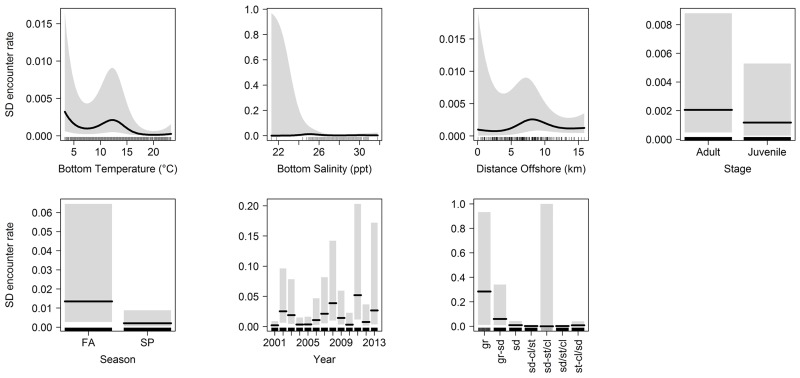
Fitted back-transformed smoothing curves for significant univariate explanatory variables in the best-fitting encounter rate probability model (1st stage). The tick marks on x-axis denote the relative density of observation. The grey envelopes represent the 95% confidence intervals. Note that the range of y-axis differs among the panels for display purposes. SD: shell disease.

**Fig 5:**
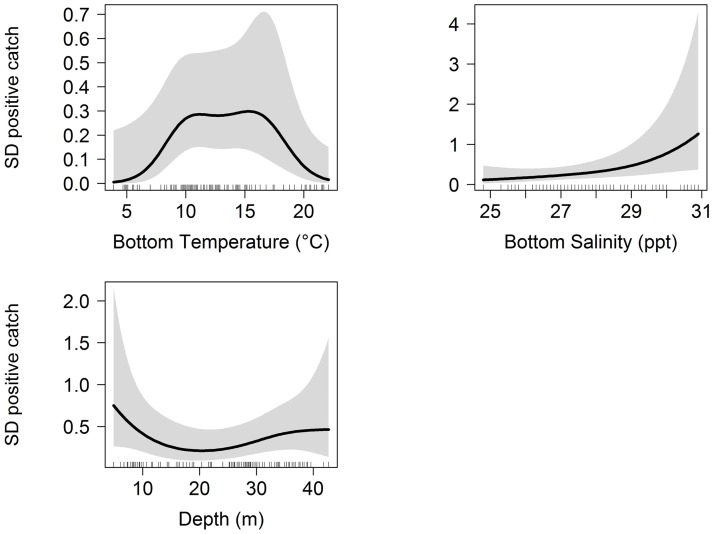
Fitted back-transformed smoothing curves for significant univariate explanatory variables in the best-fitting positive catch probability model (2nd stage). The tick marks on x-axis denote the relative density of observation. The grey envelopes represent the 95% confidence intervals. Note that the range of y-axis differs among the panels for display purposes. SD: shell disease.

Bottom temperature and bottom salinity were included in the best-fitting encounter rate probability model (Figs 4 and 5). Both abiotic variables had a significant non-linear effect on the probability of shell disease presence. The bottom temperature response curves from the best-fitting encounter rate probability model showed higher probability of shell disease presence at < 5°C and between 10–15°C, while the temperature response curve from the positive catch model showed that the relationship was dome-shaped with a peak probability of shell disease abundance between 10–15°C. Bottom salinity also showed significant effect on both shell disease encounter rate and positive catch probability, where the probability of shell disease presence peaked at ~25 ppt, while the probability of shell disease positive catch increased at higher salinity ranges (Figs 4 and 5). Distance offshore was included in the best-fitting encounter rate probability model, while depth was included in the best-fitting positive catch model (Figs 4 and 5). The distance offshore response curve from the encounter rate probability model indicates that the probability of disease presence peaked between 5–10 km (Fig 4). The probability of conditional disease abundance was lowest at approximately 20 m depth (Fig 5).

A year effect was included in the best-fitting encounter rate probability model as a significant temporal variable (Fig 4). The disease encounter rate probability per tow was the lowest in 2001, but peaked in 2011. Effects of bottom type, stage, and season were only significant for the encounter rate probability model. The highest disease encounter rate probability was associated with gravel, while the lowest encounter rate probability was associated with sand-silt/clay (Fig 4). The adult life stage (CL > 60 mm) and fall season (September-October) were also associated with higher probability of disease presence (Fig 4).

### Model fitting and validation

**Fig 6:**
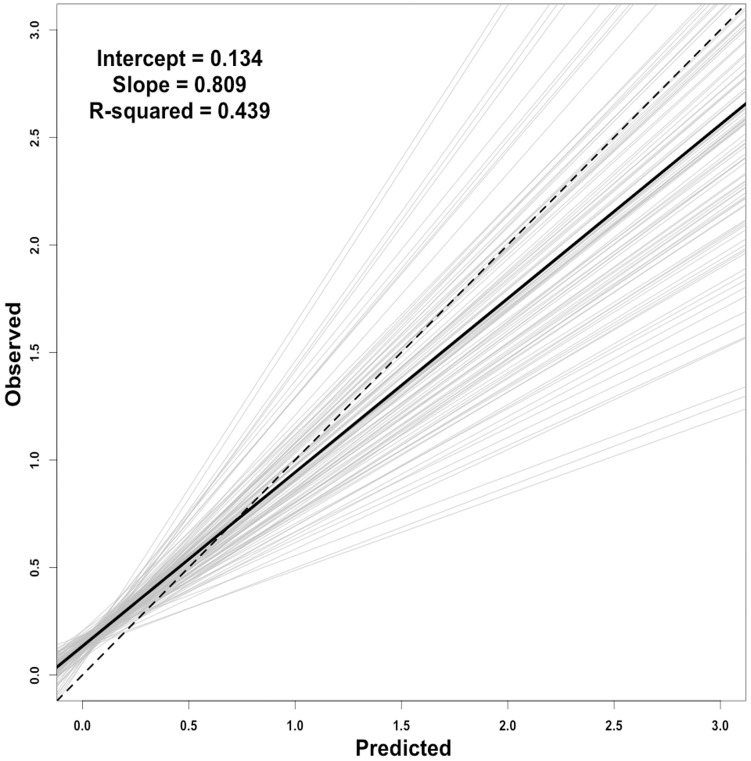
Bivariate observed versus predicted plot complemented by the graphical summary of regression analyses from 100 runs of cross-validations for the delta-generalized additive modelling (GAM) effort. The light gray lines represent 100 linear regression lines. The black line represents the mean of 100 linear regression lines. The dashed line represents the 1:1 line and an ideal model performance.

All candidate explanatory variables were observed with VIF less than 3 (Table 1), therefore multicollinearity was determined to be negligible in the model development. The best-fitting binomial GAM (1^st^ stage encounter rate probability model) explained 56.3% of the deviance, while the best-fitting negative-binomial GAM (2^nd^ stage positive catch model) explained 31.3% of the deviance (Table 2). A comparison of the mean cross-validation results with an ideal model performance (e.g. a model without prediction bias; *α* = 0, *β* = 1, and *R*^*2*^ = 1) indicated that the delta-GAM predicted the overall shell disease prevalence well (*α* = 0.134, *β* = 0.809, and *R*^*2*^ = 0.43; Fig 6). A slight bias toward over-prediction at low prevalence was observed while the degree of over-prediction increased with higher prevalence. However, the model’s predictive performance was considered to be sufficient for predicting an overall distribution of the true shell disease prevalence in this study.

**Table 2:** Comparison of full and best-fitting generalized additive model (GAM) results for the delta modeling approach.

**1st stage "Encounter Rate Probability" GAM (n = 2008)**				
**Model**	**Formula**	**edf**^a^	**Deviance explained (%)**	**AIC**^b^
Full	Size + Sex + Season + Year + Sediment Type + s(Bottom Temperature) + s(Bottom Salinity) + s(Depth) + s(Distance Offshore) + s(Longitude) + s(Latitude)	3.98 3.89 3.62 3.50 3.06 1.00	50.10	1453.08
Best-fitting	Size + Season + Year + Sediment Type + s(Bottom Salinity) + s(Distance Offshore) + s(Bottom Temperature) + s(Longitude, Latitude)	3.86 3.67 3.95 26.72	56.30	1371.03
**2nd stage "Positive Catch Probability" GAM (n = 142)**				
**Model**	**Model**	**edf**	**Deviance explained (%)**	**AIC**
Full	Size + Sex + Season + Year + Sediment Type + s(Bottom Temperature) + s(Bottom Salinity) + s(Depth) + s(Distance Offshore) + s(Longitude) + s(Latitude)	2.99 2.96 1.04 1.00 1.04 1.00	53.20	217.346
Best-fitting	s(Bottom Temperature) + s(Bottom Salinity) + s(Depth)	3.21 1.42 2.38	31.30	207.056

^a^edf: estimated degree of freedom

^b^AIC: Akaike information criterion

### Delta-GAM prediction and survey-based gravity centers of disease prevalence

**Fig 7:**
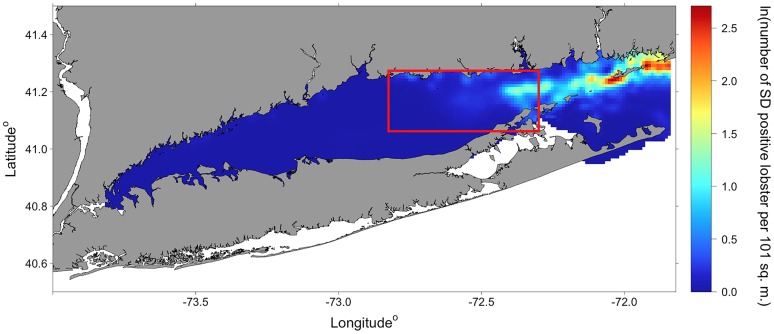
Mean spatial variation of predicted zero inflation adjusted shell disease (SD) prevalence, expressed as ln(number of SD positive lobster per 101 m^2^), for 2001–2013. The red rectangle represents the spatial domain of Figure 7b.

**Fig 8:**
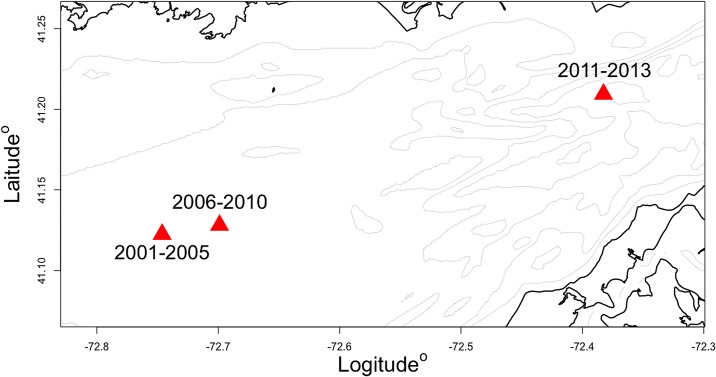
Observed inter-annual variability in shell disease gravity centers for 2001–2013.

The delta-GAM was used to generate zero inflation adjusted estimate of shell disease prevalence (per minute towing; 101 m^2^). The predicted shell disease prevalence in LIS showed a ‘high-east: low-west’ spatial pattern (Fig 7). The delta-GAM predicted high disease prevalence in the shallow waters on the southwestern and northeastern sides of Fishers Island in northeastern LIS. The survey-based gravity centers of shell disease shifted northeastward during 2001–2013 in the area between 72.8:72.3° W and 41.1:41.25° N (Fig 8); however, the survey-based gravity centers did not coincide spatially with the predicted disease hotspots.

## Discussion

### Ecological interpretation of model outputs

The delta-GAM developed in this study identified a high concentration of shell disease prevalence in northeastern LIS (Fig 7). A similar pattern has been documented in other studies, although its drivers remain difficult to identify. However, bottom water temperature has been frequently cited as one major contributor to shell disease occurrence [1,63]. Because eastern LIS has had higher rates of temperature increase and higher mean maximal monthly temperatures than western LIS [28], this could be influencing the patterns we describe. Eastern LIS is also known to have higher levels of contaminants such as PCBs, pesticides, and metals than other regions of the Sound, which have also been noted as potential contributors to various lobster diseases [1,64].

Other potential causes of shell disease seem to be distributed paradoxically to the east-high, west-low spatial prevalence patterns. For example, shell diseased symptoms occur when the loss of shell material exceeds its natural deposition [8], therefore it is expected that shell disease would coincide with areas with high concentrations of alkylphenols, which inhibit shell growth [65]. However, Jacobs et al. [66] found that levels of alkylphenol contamination was highest in lobsters from western LIS, where observed disease prevalence is generally lowest. Similarly, presumably stress-inducing hypoxia increases in severity from east to west, in opposition to the shell disease prevalence documented here [67].

Potential insights into disease etiology in LIS can also be gained by evaluating univariate explanatory variables individually. For instance, the response curves from best-fitting binomial and negative-binomial GAMs were generally in agreement with existing literature related to habitat tolerance of American lobster with regard to bottom temperature, bottom salinity, depth and sediment type [7,31,39,68–71], indicating shell disease occurrence often coincides with optimal or near-optimal lobster habitat conditions. For instance, Tanaka and Chen [31] identified suitable salinity for lobster in LIS is between 21 and 30.4 ppt, which is also contained the salinity range where shell disease is found (Fig 4). These results are unexpected given past research (e.g., Tlusty et al. [8]) suggesting that environmentally-induced physiological stress is a precursor to shell disease incidence. However, this pattern could be explained by an increased propensity for infected individuals to move away from stressful conditions found in suboptimal environments, due to the costs they are incurring while subjected to stressful conditions.

Water temperature has been previously identified as a significant contributor to shell disease occurrence [1,63]. The significant, nonlinear relationship between bottom water temperature and shell disease encounter rate probability we documented (Fig 4) is likely reflective of lobsters’ varied molting rate at different temperatures and ability to molt out of a moderately infected shell [72]. For instance, encounter rate probability peaks between 10–14°C, when disease progression may be outpaced by molting rates. Similarly, the reduction in prevalence toward 20°C could be attributable to molting rate exceeding disease progression. The increasing presence of shell disease in fall as indicated by our model coincides with previous studies performed in eastern LIS where disease prevalence increased through the summer and into fall as waters warmed [73,74] as well as near Massachusetts where the highest concentration of shell disease in the study area correlated with cumulative periods of time where water temperatures exceeded 20°C [63].

This model further reinforces the likely role of demographic characteristics to shell disease susceptibility. Because juveniles tend to molt more frequently, less time is allowed for shell disease to become established before a shell is molted. Therefore, the significance of age in our model are likely due to extended intermolt durations for large individuals [72]. Ovigerous females have often been found to have a higher incidence of shell disease than either males or non-reproductive females due to delayed molting cycles [27,75]; however, our model did not detect a significant effect of sex. We attribute this result to the concatenation of samples taken throughout the year, which may mask the effects of higher prevalence for females during egg-bearing times of the year when molting is postponed.

### Model implications and limitations

For reasons of logistical rationality and simplicity, monitoring of marine species is conducted based on a spatiotemporal scale relevant to observers, not marine species [76]. This bias, due to differences between the stratification strategies employed by the observer and marine species, results in disease presence, origins and spread often remaining undetected [16]. In this study, the delta-GAM predicted a significant hotspot of lobster shell disease in the non-surveyed area in the northeast of the LIS, which did not coincide with observed shell disease gravity centers. The model-based disease probability map can be used to generate hypotheses about exposure for further investigation by overlaying with maps of potential anthropogenic pollution sources and areas where lobsters are under prolonged environmental-stress. Association of the marine disease to surrounding abiotic and biotic factors in many cases is poorly understood. The delta-GAM approach developed in this study can enhance our understanding of continuing lobster shell disease threats and monitoring effort by (1) quantifying the significance and association of environment and host characteristics in lobster shell disease prevalence, and (2) developing a parsimonious statistical modeling framework to predict the spatial distribution of shell disease prevalence from zero-inflated observations.

Our approach has a number of potential limitations. While one of the objectives of this study was to develop a simple, parsimonious modeling framework to complement both descriptive and predictive research priorities, GAM is a data driven approach that is often limited by the data available for model calibration. For example, a p-value of 0.05 was used as cut off for statistically significant associations, but it is important to acknowledge that some key covariates (e.g. host sex) may be determined not statistically significant and excluded simply due to; (1) the relatively small number of diseased lobsters in the original data, and (2) significant associations exerted by abiotic (e.g. bottom temperature) and spatial variables (e.g. latitude *longitude interaction) “masking” the weaker associations of these biological variables. The location variables (i.e. latitude and longitude) were used to capture the localized effects [55,77]; however, provided that the data are available, incorporating key variables such as pollution, pH level, surface chlorophyll, hypoxia frequency, and population memory would likely allows us to further tune the delta-GAM to be a more comprehensive management tool [40,78]. Further improvements could be made by applying models that explicitly account for progression of disease prevalence over time, integrate both measured and unmeasured covariates, and include the consideration of spatial and temporal autocorrelation [40,79]. However, while such an advanced model may yield better predictive performance, other aspects of model performance should also be considered (e.g. ecological realism as well as model usability to non-expert stakeholders)[56]. It is also important to acknowledge that the best-fitting models identified in this study were developed for specificity over generality to allow interpolation in LIS (i.e. filling in the gaps in survey data and describing known disease distributions), and the model outcomes in the area outside of LIS should not be considered. A simpler model will be required to make more general but robust extrapolation through space or time [56,80].

Finally, distinction and trade-off between empirical-based statistical modeling approaches (e.g., GAM) and process-based mechanistic modeling approaches (e.g., agent-based model) should be addressed explicitly [56]. In an epidemiological context, the strength of a statistical modeling approach lies in its ability to provide a mathematical basis for hypothesized associations between observed disease prevalence and environmental factors [18], while mechanistic modeling approaches can simulate underlying processes driving the disease prevalence [19]. As for the trade-off, both approaches are subject to specific sources of uncertainty. For instance, where empirical-statistical models are unable to incorporate source-sink processes, process-mechanistic models are unlikely to capture the true complexity of ecosystems [81]. The empirical-based statistical modeling framework presented in this study represents a first step toward comprehensive modeling efforts to better understand the complex epizootic disease dynamics. For example, GAM can be used to incorporate ecological information associated with the geographical distribution and habitat suitability of diseased lobsters for more mechanistic approaches [82], which can potentially predict the habitat-dependent environmental impact on shell disease dynamics more accurately.

### Management implications

Harvell et al. [16] identified several key marine disease management priorities, which can enhance the research, monitoring and management of emerging and continuing marine disease threats. These include pinpointing the role of biotic and abiotic factors in disease spread, developing forecasting models for outbreaks that are sensitive to environmental and climatic factors, and implementing ecosystem-based surveillance programs for emerging marine diseases. The combination of empirical data and modeling presented here aims to address these management priorities and provide a valuable tool for the management of inshore and offshore lobster fisheries, which were the highest valued commercial fishery in 2014, worth in excess of half a billion dollars [12]. The approach can be used to guide decision-making in monitoring and management of lobster shell disease. Ultimately, our findings will provide policy-relevant information for effective ecosystem-based disease surveillance programs, which could be of value for the fisheries.

The modeling approach described here also provides the framework from which similar models could be developed for other marine organisms and marine diseases in the U.S. and international fisheries. Groner et al. [83] call for *“data driven forecasting and predictive modeling”* to adaptively manage emerging marine diseases. The delta-GAM outputs presented in this study can potentially facilitate an effective ecosystem-based management of the commercially important fisheries that are under disease threat. If data are available, the model can also investigate the impact of anthropogenic agents and pathogens. The success of these actions are dependent upon the major environmental risk factors for the disease being known and that the relevant environmental data are of the appropriate temporal and spatial resolution for the organism under investigation [83]. As the origins and spread of most marine diseases are poorly known [16], the modeling approach described in this study renders a novel first step towards identifying the potential biotic and abiotic conditions contributing to marine diseases [83]. Furthermore, through establishment of a framework whereby environmental contributions to disease presence and prevalence may be identified, this modeling approach can potentially provide reliable information for future mechanistic models that may provide the basis for models more predictive in nature, a need highlighted in recent work on marine disease [28,83].

Fisheries managers require flexible low-cost tools to help deal with the emerging threat of marine disease. This need is exacerbated by the increasing likelihood of abrupt, nonlinear environmental and climatic changes [83]. Management strategies, such as closures to reduce fishing morality in order to help restore the stock at broad spatial scales can be costly to implement and to those whose livelihoods are dependent on the managed marine species. In addition, these ‘broad brush’ approaches may impact areas not impacted by disease, thus increasing their cost and impact unnecessarily. Reliable and up-to-date maps of marine diseases, like those provided by this modeling approach, can enhance the monitoring of emerging and continuing marine disease threats by improving the geographical targeting and cost-effectiveness of existing sampling programs which are often limited by logistical hurdles (e.g. cost, resources). Given the increasing uncertainty in the health of the marine resources upon which people rely driven by linear long term climate trends and more abrupt climatic perturbations, the types of low-cost tools that leverage existing monitoring datasets (e.g. trawl surveys) like the model outlined here can provide essential information in managing wild harvest fisheries that are constantly under disease threats.

## Supporting information

S1 Table The set of candidate models identified in the delta modeling approach. (DOCX)
