# Supplementary material for: A statistical model for monitoring shell disease in inshore lobster fisheries: A case study in Long Island Sound
Source: PLoS One. 2017 Feb 14;12(2):e0172123. doi: 10.1371/journal.pone.0172123 (PMC5308772; doi:10.1371/journal.pone.0172123)
Supplement: S1 Table — (DOCX) [file pone.0172123.s001.docx]

**S1 Table. The set of candidate models identified in the delta modeling approach.**

| **1st stage "Encounter Rate Probability" GAM (n = 2008)** | | |  | | | |  | | |
| --- | --- | --- | --- | --- | --- | --- | --- | --- | --- |
| **Model** | **Formula** | **edf** | | | **Deviance explained (%)** | **AIC** | | **Δ AIC** |  |
| Full | Size + Sex + Season + Year + Sediment Type + s(Bottom Temperature) + s(Bottom Salinity) + s(Depth) + s(Distance Offshore) + s(Longitude) + s(Latitude) | 3.97 3.79 3.21 3.71 2.98 1.00 | | | 50.1 | 1453.08 | |  |  |
| Candidate | Size + Season + Year + Sediment Type + s(Bottom Temperature) + s(Bottom Salinity) + s(Depth) + s(Distance Offshore) + s(Longitude) + s(Latitude) | 3.97 3.79 3.21 3.71 2.98 1.00 | | | 50.1 | 1451.10 | | -1.98 |  |
| Candidate | Size + Season + Year + Sediment Type + s(Bottom Temperature) + s(Bottom Salinity) + s(Depth) + s(Distance Offshore) + s(Longitude, Latitude) | 3.95 3.80 2.68 3.81 25.83 | | | 56.5 | 1370.29 | | -80.81 |  |
| Best-fitting | Size + Season + Year + Sediment Type + s(Bottom Temperature) + s(Bottom Salinity) + s(Distance Offshore) + s(Longitude, Latitude) | 3.86 3.67 3.95 26.72 | | | 56.3 | 1371.03 | | +0.74 |  |
|  |  |  | | |  |  | |  |  |
| **2nd stage "Positive Catch Probability" GAM (n = 142)** | | |  |  |  |  |  |  |  |
| **Model** | **Model** | **edf** | | | **Deviance explained (%)** | **AIC** | | **Δ AIC** |  |
| Full | Size + Sex + Season + Year + Sediment Type + s(Bottom Temperature) + s(Bottom Salinity) + s(Depth) + s(Distance Offshore) + s(Longitude) + s(Latitude) | 3.33 3.12 1.03 1.03 1.06 1.06 | | | 54.7 | 230.38 | |  |  |
| Candidate | Size + Sex + Season + Year + s(Bottom Temperature) + s(Bottom Salinity) + s(Depth) + s(Distance Offshore) + s(Longitude) + s(Latitude) | 2.50 1.25 2.12 1.74 3.15 1.25 | | | 46.2 | 228.11 | | -2.27 |  |
| Candidate | Size + Season + Year + s(Bottom Temperature) + s(Bottom Salinity) + s(Depth) + s(Distance Offshore) + s(Longitude) + s(Latitude) | 3.03 2.64 2.15 1.00 1.00 1.85 | | | 48.7 | 225.56 | | -2.55 |  |
| Candidate | Size + Season + s(Bottom Temperature) + s(Bottom Salinity) + s(Depth) + s(Distance Offshore) + s(Longitude) + s(Latitude) | 3.02 2.05 2.53 1.00 1.00 1.00 | | | 34.4 | 215.62 | | -9.94 |  |
| Candidate | Size + Season + s(Bottom Temperature) + s(Bottom Salinity) + s(Depth) + s(Distance Offshore) + s(Latitude) | 3.04 1.93 2.44 1.00 1.00 | | | 33.8 | 213.68 | | -1.94 |  |
| Candidate | Size + s(Bottom Temperature) + s(Bottom Salinity) + s(Depth) + s(Distance Offshore) + s(Latitude) | 3.05 1.93 2.54 1.00 1.00 | | | 33.9 | 211.71 | | -1.96 |  |
| Candidate | Size + s(Bottom Temperature) + s(Bottom Salinity) + s(Depth) + s(Distance Offshore) | 3.04 2.01 2.52 1.00 | | | 33.7 | 209.74 | | -1.97 |  |
| Candidate | Size + s(Bottom Temperature) + s(Bottom Salinity) + s(Depth) | 3.04 1.89 2.45 | | | 32.6 | 208.68 | | -1.06 |  |
| Best-fitting | s(Bottom Temperature) + s(Bottom Salinity) + s(Depth) | 3.21 1.42 2.38 | | | 31.3 | 207.06 | | -1.62 |  |

^a^edf: estimated degree of freedom

^b^AIC: Akaike information criterion
